# Malignant transformation of plexiform neurofibroma to MPNST while on MEK inhibitor

**DOI:** 10.1093/noajnl/vdab033

**Published:** 2021-02-23

**Authors:** Samir Fasih, Suganth Suppiyah, Jane Barron, Carolina Barnett-Tapia, Roger Avery, Brendan Dickson, Peter Ferguson, Carol Swallow, Gelareh Zadeh, Abha A Gupta

**Affiliations:** 1 Division of Medical Oncology, Princess Margaret Cancer Center, University of Toronto, Toronto, Ontario, Canada; 2 Division of Neurosurgery, University Health Network, University of Toronto, Toronto, Ontario, Canada; 3 Discipline of Laboratory Medicine, Memorial University of Newfoundland, St. John's, Newfoundland and Labrador, Canada; 4 Department of Surgery, Memorial University of Newfoundland, St. John's, Newfoundland and Labrador, Canada; 5 Department of Pathology, Mount Sinai Hospital, University of Toronto, Toronto, Ontario, Canada; 6 Department of Orthopedic Surgery, Mount Sinai Hospital, University of Toronto, Toronto, Ontario, Canada; 7 Department of General Surgery, Mount Sinai Hospital, University of Toronto, Toronto, Ontario, Canada

Neurofibromatosis type 1 (NF1) is a neurocutaneous tumor predisposition syndrome resulting in the development of multiple peripheral nerve sheath tumors, or, plexiform neurofibromas (PN), some of which can transform into high-grade sarcomas, called malignant peripheral nerve sheath tumors (MPNST).^1^ NF1-associated MPNST are highly aggressive sarcomas with a 5-year survival ranging from 20% to 50%, as these tumors have high rates of local recurrence and distant metastasis.^2^ Oral selective mitogen-activated protein kinase (MAPK) kinase (MEK) inhibitors have shown impressive clinical activity in children and adolescents with PN with durable and significant decrease in tumor volume.^3–6^ Exploration of the safety and efficacy of MEK inhibitors in adults is also emerging.^7^ We report 2 young adults with NF1 who developed MPNST within a few months of starting MEK inhibitors.

A 33-year-old male with NF1 was being followed for a cervical spine PN since age 26. He had previously undergone surgery (2012) and radiation (2016) for this lesion, but then due to recurrent pain, repeat imaging and biopsy were done 02/2019, confirming PN. Trametinib (2 mg once daily) was started 04/2019 with significant improvement in limb weakness. He continued drug until 30/6/2019, at which time he developed cervical cord compression leading to respiratory compromise (Figure 1). Repeat MRI showed complete cord effacement and filling the canal with tumor; patient died of respiratory failure on 2/7/2019, 3 months after starting Trametinib. Partial autopsy confirmed malignant transformation to MPNST.

**Figure 1.**
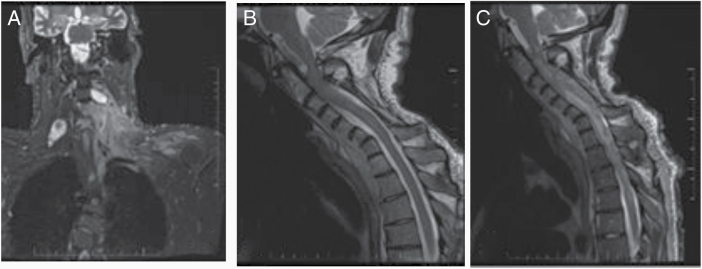
Imaging of Case 1 (A, B, C). (A) Case 1. MRI June 2018, shows neurofibroma in neck; (B) Case 1. MRI April 2019, normal cord; (C) Case 1. MRI June 2019 complete cord effacement and filling of the spinal canal with tumor.

The second patient was a 25-year-old female with NF1, who had numerous stable PN. One of the intrathoracic lesions caused chronic dysphagia and pain for which she started Trametinib 10/2018 (2 mg once daily). In 06/2019 she had increasing pain and size of the left lower abdominal wall and right buttock lesions. MRI showed marked interval enlargement of both lesions. Trametinib was stopped on 25/6/2019; both lesions were resected and demonstrated high-grade MPNST. Patient developed pulmonary metastases 7/2019 and died from disease 12/2019.

Chromosomal copy number variation profiles generated from raw methylation data demonstrated that both cases had numerous chromosomal gains and losses, reminiscent of chromothripsis. Notably, the *NF1* gene locus on chromosome 17q and *CDKN2A/B* locus on chromosome 9p was lost in both samples, consistent with other NF1-associated MPNSTs. In addition, unsupervised clustering with t-distributed stochastic neighbor embedding (t-SNE) of the top 10,000 most variably methylated probes demonstrate that both cases share a similar methylation signature to normal NF1-associated MPNSTs

We have demonstrated 2 adults undergoing transformation of their PN into MPNST despite being on MEK-inhibitor therapy. The impact of MEK inhibitors for young patients has been insurmountable, accounting for the FDA approval for selumetinib for the indication of PN in children and adolescents with NF1. The 2 cases we report herein were of age 33 and 25 years, and fall within the median age for MPNST transformation.^2^ Both had begun drug within 6–8 months of presenting with MPNST, with demonstration of relative PN stability prior to starting drug. Starting the MEK inhibitor did not prevent malignant transformation.

With the death of these 2 young adult patients, we suggest that MEK inhibitors should only be used in adult NF1 patients who have close radiographic and clinical surveillance of all PN lesions. Further analysis is required to understand the impact of MEK inhibition on malignant transformation of PN in adults with NF1.

## Notes

**Authorship Statement.** Manuscript preparation—all; laboratory analysis—S.S., G.Z.
